# Seasonal variation of microbiota composition in *Anopheles gambiae* and *Anopheles coluzzii* in two different eco‐geographical localities in Cameroon

**DOI:** 10.1111/mve.12583

**Published:** 2022-05-17

**Authors:** Maurice Marcel Sandeu, Claudine Grâce Tatsinkou Maffo, Nsa Dada, Flobert Njiokou, Grant L. Hughes, Charles S. Wondji

**Affiliations:** ^1^ Department of Medical Entomology Centre for Research in Infectious Diseases (CRID), LSTM Research Unit Yaoundé Cameroon; ^2^ Department of Microbiology and Infectious Diseases, School of Veterinary Medicine and Sciences University of Ngaoundéré Ngaoundéré Cameroon; ^3^ Department of Animal Biology and Physiology, Faculty of Science University of Yaoundé 1 Yaoundé Cameroon; ^4^ Faculty of Science and Technology Norwegian University of Life Science Aas Norway; ^5^ Tropical Infectious Disease Research Center University of Abomey‐Calavi Cotonou Benin; ^6^ Departments of Vector Biology and Tropical Disease Biology Centre for Neglected Tropical Diseases, Liverpool School of Tropical Medicine Liverpool UK; ^7^ Department of Vector Biology Liverpool School of Tropical Medicine, Pembroke Place Liverpool UK

**Keywords:** 16S rRNA gene amplicon sequencing, *Anopheles coluzzii*, *Anopheles gambiae ss*, mosquito microbiota, seasonality

## Abstract

Understanding the environmental factors affecting the microbiota in malaria vectors may help in the development of novel vector control interventions, similar to paratransgenesis. This study evaluated seasonal and geographical variations in the microbial community of the two major malaria vectors. Adult *Anopheles* mosquitoes were collected across two different eco‐geographical settings in Cameroon, during the dry and wet seasons. DNA was extracted from the whole individual mosquitoes from each group and processed for microbial analysis using Illumina Miseq sequencing of the V3‐V4 region of the 16S rRNA gene. Data analysis was performed using QIIME2 and R software programs. A total of 1985 mosquitoes were collected and among them, 120 were selected randomly corresponding to 30 mosquitoes per season and locality. Overall, 97 bacterial taxa were detected across all mosquito samples, with 86 of these shared between dry and wet seasons in both localities and species. There were significant differences in bacterial composition between both seasons, with a clear separation observed between the dry and wet seasons (PERMANOVA comparisons of beta diversity, Pseudo‐*F* = 10.45; *q*‐value = 0.01). This study highlights the influence of seasonal variation on microbial communities and this variation's impact on mosquito biology and vectorial capacity should be further investigated.

## Graphical abstract

1. This study evaluated seasonal and geographical variations in the microbial community of the two major malaria vectors (*Anopheles gambiae* and *Anopheles coluzzii*).
2. Overall, 97 bacterial taxa were detected across all mosquito samples, with 86 of these shared between dry and wet seasons in both localities and species.
3. Significant differences in bacterial composition between both seasons were identified, with a clear separation observed between the dry and wet seasons (Beta diversity, Pseudo‐*F* = 10.45; *q*‐value = 0.01).

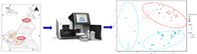

## BACKGROUND

Mosquitoes are among the most investigated group of insects because of their remarkable role in the transmission of various parasites and pathogens to humans, domestic animals and wildlife (Muturi et al., 2018). They serve as vectors of several pathogens that cause diseases such as malaria, dengue, yellow fever, Zika, West Nile fever and chikungunya. Among these, malaria alone accounts for around 229 million cases and about 400,000 deaths every year, globally (Muturi et al., 2018). Malaria is caused by *Plasmodium* parasites and is transmitted to humans by female *Anopheles* mosquitoes including *An. coluzzii*, *An. gambiae* ss and *An. funestus* as the main malaria vector in Cameroon (Antonio‐Nkondjio et al., 2019). Considering the lack of an effective vaccine and increasing resistance to chemical insecticides, efforts to find innovative methods for controlling malaria through a better understanding of factors that influence both vector competence and vectorial capacity are urgently needed (Karimian et al., 2019; Naseri‐Karimi et al., 2015; Shaw & Catteruccia, 2019; Wang et al., 2017).

Research on mosquito‐microbiome interactions may lead to new tools for mosquito and mosquito‐borne disease control. Mosquitoes have been shown to harbour microbial gut communities that enhance their fitness by contributing to digestion, nutrition, reproduction or providing protection from pathogens (Douglas, 2014). The mosquito gut microbiota is mainly acquired from the environment (Strand, 2018; Wang et al., 2017) and its composition is largely influenced by its aquatic breeding environment (Coon et al., 2014; Gimonneau et al., 2014). In addition, the microbiota composition is highly dynamic, varying greatly with species and even among individuals of the same species (Hegde et al., 2018; Muturi et al., 2016; Osei‐Poku et al., 2012; Scolari et al., 2019), stage of mosquito development, and geographical areas (Duguma et al., 2019; Krajacich et al., 2017; Minard et al., 2017; Tchioffo et al., 2015). These variations of microbiota composition between field individual mosquitoes may partly explain the variability in mosquito infection levels in the field (Rosenberg, 2008). Some studies have shown that there is minor seasonal variation in *An*. *gambiae s.s*. mosquito microbiota from the forest‐savannah regions with perennial larval sites during the dry season in Ghana, but that limited differences were found within *An*. *coluzzii* between seasons and locations (Akorli et al., 2016). Others revealed that microbiota composition was also different between the seasons and localities in Mali (Krajacich et al., 2017) and that the composition of microbiota can be shaped by host sampling location (Muturi et al., 2018). Also, spatial and temporal environmental variations altering microbiota have been shown in fruit flies (Corby‐Harris et al., 2007). Although the mosquito microbiota composition is largely influenced by their aquatic stage breeding site, all the exact factors defining the structure of adult mosquito microbiota are currently unknown (Gendrin & Christophides, 2013) thus the extension of the microbiome study to other localities and *Anopheles* species could be used for malaria control.

Identifying the effect of environmental variables on the microbiome and widely characterizing the native microbiota composition of mosquito vectors is important for developing and deploying symbiotic control strategies. However, there has been no work to date on the spatio‐temporal variability in the microbiota of Cameroonian mosquitoes. Therefore, this study aimed to evaluate the composition and structure of the microbiota in *An. coluzzii* and *An. gambiae*, two main malaria vectors in Cameroon, were collected from different locations during dry and wet seasons. In addition, this study aims at identifying potential natural bacteria symbionts that can be used to develop novel approaches for mosquito control.

## MATERIALS AND METHODS

### Study sites and mosquito collection

This study was conducted in two localities in Cameroon, namely Gounougou (Northern region 9°03′00″N, 13°43′59″E) and Bankeng (Centre region, 4°40′26.4″N, 12°22′30″E.) (Figure S1). These are two (2) different eco‐geographical areas where the occurrence of *Anopheles* mosquitoes has already been demonstrated (Elanga‐Ndille et al., 2020; Fadel et al., 2019). Gounougou is Sudano‐Sahelian, while Bankeng is a tropical forested area. Gounougou is a village in the commune of Lagdo located in the department of Bénoué, Northern Region. It lies along the River Bénoué, in an area with cotton farming and rice cultivation. The climate of Gounougou is characterized by a short rainy season from May to September (mean annual rainfall of 900–1000 mm) and a long dry season from October to April. Bankeng is a small village in the department of Haute Sanaga, an area of tropical forest. This village is also a rice‐growing area with watercourses (along the Sanaga River). The climate is characterized by a short dry season from December to March and a long rainy season from April to November (mean annual rainfall of 1000–2000 mm).

Mosquito collections were conducted in both study sites during the dry season from December 2018 to January 2019, and during the wet season in August 2019. Indoor resting female mosquitoes were collected between 06:00 am and 09:00 am following verbal consent from the village chief and each household representative. Mosquitoes were collected using Prokopack electrical aspirators (John W. Hook, Gainesville, FL). The collected mosquitoes were kept in individual tubes and subsequently transported to the insectary at the Centre for Research in Infectious Diseases (CRID), Yaoundé for morphological identification using keys for Afro‐tropical anopheline mosquitoes (Gillies & De Meillon, 1968). In the end, these samples collected were used to characterize *Anopheles* species composition in both localities during both seasons, and the downstream microbiota characterization was focused on the predominant mosquito species or taxa in each location.

### *Sample processing, DNA extraction, and molecular identification of* Anopheles s.l *species*

A total of 120 individual mosquito samples from the two localities during the two seasons of collection corresponding to 30 randomly selected individuals per location and season were processed for microbial analysis. Prior to genomic DNA extraction, individual adult female mosquitoes were surface sterilized by washing them in 70% ethanol for 5 min and then rinsing twice with sterile distilled water in order to remove superficial bacteria to avoid external contamination. Genomic DNA was extracted from whole individual mosquitoes using a Genejet extraction kit following the manufacturer's recommendations. Isolated DNA was reconstituted in 100 μl of Elution buffer and two aliquots of 50 μl each were prepared and stored at −20°C until further processing. One 50 μl aliquot of the resulting DNA isolate was utilized to build a microbiota library for Illumina sequencing. The other 50 μl aliquot of the DNA isolate was used for molecular identification of the members of the *An. gambiae s.l*. complex using the Short Interspersed Elements (SINE) (Santolamazza et al., 2008). Three negative controls, in which the extraction procedure was performed without adding any mosquito template were included to check for microbial contamination.

### 16S rRNA gene amplification, library preparation, and 16S rRNA sequencing

All the 120 selected DNA samples were sent to Polo d'Innovazione di Genomica, Genetica e Biologia (Infravec) for sequencing using the Illumina Miseq system. Three negative controls comprised of the water blanks and one positive control comprised of a mock bacterial community were processed similarly. The PCR amplification and sequencing procedures targeting the V3‐V4 hypervariable region of bacterial and archaeal 16S rRNA gene were performed following the Illumina 16S Metagenomic Sequencing Library Preparation Guide (Part # 15044223 Rev. B) using the Nextera XT Index Kit and 16S Amplicon PCR Forward Primer.

5′TCGTCGGCAGCGTCAGATGTGTATAAGAGACAGCCTACGGGNGGCWGCAG‐3′ and 16S Amplicon PCR Reverse Primer 5′‐GTCTCGTGGGCTCGGAGATGTGTATAAGA GACAGGACTACHVGGGTATCTAATCC‐3′ (Klindworth et al., 2013).

For the Illumina library preparation, a two‐step PCR amplification process was used. The first involved amplification of the bacterial 16S rRNA gene V3‐V4 for each sample. The 25 μl reaction contained 2.5 μl DNA (5 ng/μl), 5 μl each primer (1 μM), and 12.5 μl 2x KAPA HiFi HotStart Ready mix. The cycling conditions consisted of an initial denaturation at 94°C for 3 min, followed by 25 cycles of denaturation at 94°C for 30 s, annealing at 55°C for the 30 s, elongation at 72°C for 30s with a final elongation at 72°C for 5 min. The expected sizes of the PCR products were verified by running 1 μl of the PCR product on a Bioanalyzer DNA 1000 (expected size ~550 bp). PCR products were cleaned up using AMPure XP beads at 1x sample volume to purify the 16S V3 and V4 amplicon away from free primers and primer‐dimer species. A second PCR was carried out to incorporate dual Illumina adapter sequences using the Nextera XT Index Kit. PCR reactions were performed in a total volume of 25 μl containing 5 μl DNA, 5 μl each primer (Nextera XT Index Primer i7 and i5), and 25 μl 2x KAPA HiFi HotStart Ready‐mix and 10 μl PCR Grade water. The amplification program consisted of an initial denaturation step at 95°C for 3 min, followed by 8 cycles each consisting of denaturation at 94°C for 30 s, annealing at 55°C for 30 s, elongation at 72°C for the 30 s and final elongation at 72°C for 5 min. AMPure XP beads at 4 pM were used to clean up the final library. The resulting libraries were validated using the Fragment Analyser (High Sensitivity Small Fragment Analysis Kit) to check size distribution. The concentration of library samples was defined on the basis of the Qubit® 3.0 Fluorometer quantification and average library size. For Cluster generation and sequencing, indexed DNA libraries were normalized to 4 nM and then pooled in equal volumes. The pool was loaded at a concentration of 4 pM onto an Illumina Flow cell standard with 20% of Phix control. The samples were subsequently sequenced using the Illumina V2 chemistry, 2x250 bp paired‐end run.

### Sequence data processing and generation of amplicon sequence variants (ASV_S_ ) table

Raw sequence data derived from the sequencing process were demultiplexed and transferred into FASTA files for each sample, along with sequencing quality files. The resulting data were processed and analysed using the Quantitative Insights Into Microbial Ecology (QIIME2 v. 2020.2) pipeline as follows. Primers and adapter sequences were removed using the QIIME2 v.2020.2 cutadapt plugins v.2020.2 (Martin, 2011). The divisive amplicon denoise algorithm DADA2 (Callahan et al., 2016) plugin in QIIME2 was used to denoise sequence reads; this step filters out noise and corrects errors in marginal sequences, removes chimeric sequences and singletons, merges paired‐end reads, and finally dereplicates the resulting sequences, resulting in high‐resolution amplicon sequences variants (ASVs) for downstream analysis. Using the denoise‐paired command, the DADA2 options passed were trunc_len_f: 245, trunc_len_r: 245. The ASVs were further filtered to remove ASVs associated with the negative control and any reads assigned to PCR control were also filtered using the filtered taxa plugin of QIIME2. In addition, ASVs with a minimum frequency of 200 were removed. Table S1 shows sequencing reads and ASVs summary statistics.

### Diversity indices

Analysis of microbial diversity was described within (alpha diversity) and between (beta diversity) samples. The Shannon diversity index and Observed features alpha diversity indices were calculated to estimate the inter‐individual variation of bacterial diversity in different localities during dry and wet seasons. The Observed ASVs metric was used to estimate the number of unique ASVs (or richness) present within each mosquito, while the Shannon diversity index was used to estimate both ASVs richness and evenness. To establish whether alpha diversity differs across mosquito species per season all samples were rarified to a depth of 1000 ASVs per sample, which was sufficient to capture the typical low microbiota diversity in individual mosquitoes. The resulting average Shannon and Observed indices were compared between species collected from two different localities for both seasons' using pairwise the Kruskal–Wallis tests with Benjamini‐Hochberg false discovery rate (FDR) corrections for multiple comparisons.

To compare the differences in microbiota composition and structure between mosquito groups based on measures of distance or dissimilarity, the Bray–Curtis dissimilarity indices were computed with or without rarefaction, and the resulting indices were compared between samples using pairwise PERMANOVA tests (999 permutations) with FDR corrections. There were no discernable differences between the results of rarefied and non‐rarefied data. Thus, results of Bray–Curtis dissimilarity indices using rarefied data were visualized by Principal Co‐ordinates Analysis (PCoA) plots in R (Ligges, 2009) and the non‐metric multidimensional scaling (NMDS) using the phyloseq R package (Mcmurdie & Holmes, 2013). Significance for both pairwise analyses was set to *q* < 0.05 (i.e., post FDR *p*‐value corrections).

### Taxonomic analysis and differentially abundant microbial taxa

Taxonomic analysis of ASVs was performed using QIIME2's pre‐trained Naïve Bayes classifier (Zhang & Su, 2004) and the q2‐feature‐classifier plugin (Bokulich et al., 2018). Before analysis, the classifier was trained on the QIIME‐compatible 16S SILVA reference (99% identity) database v.128 (Quast et al., 2012), and using the extract‐reads command of the q2‐feature‐classifier plugin, the reference sequences were trimmed to the V3‐V4 region (450 bp) of the 16S rRNA gene. The resulting relative abundance tables of annotated ASVs were exported into R and ggplot2 v.4 (Wickham, 2011) and used to generate stacked bar plots to visualize the relative abundance of bacterial taxa across individual samples in each group of mosquitoes (*An. gambiae*‐Dry, *An. gambiae*‐Wet, *An. coluzzii*‐Dry and *An. coluzzii*‐Wet). The relative abundance of the main phyla, main family, and the main genera (median relative abundance > 0.1%) were calculated. The detection of any inter‐individual variation was estimated using the relative abundance of main bacteria genera with the median relative abundance.

The list of bacterial genera, as derived from ASVs, in each sample and group of samples was compared using Venn diagrams. These Venn diagrams were created using an online tool (http://bioinformatics.psb.ugent.be/webtools/Venn/). Differentially abundant microbial taxa across mosquito species collected from different localities in both seasons were identified using QIIME2's analysis of the composition of microbiota (ANCOM) (Mandal et al., 2015) plugin. The cut‐off for differential abundance was set to an effect size of log F ≥ 20 and W ≥ 20, that is, a taxon was differentially abundant across species per season or localities if the ratio of its abundance to those of at least 20 other taxa (25% of all included taxa) differed significantly across species per season or localities. Prior to each analysis, ASVs frequency data were normalized by log10 transformation following the addition of pseudocounts of 1. All other parameters were set to default. The resulting statistic, W, and its default cut‐off were used to identify differentially abundant features between the seasons and between the localities. Statistical analyses were conducted using non‐parametric Kruskal–Wallis and Mann–Whitney tests. Finally, this analysis allowed us to explore the flux of bacterial symbionts acquired between two different species collected from both localities and seasons.

## RESULTS

### Anopheles *species composition and abundance*

A total of 1985 mosquitoes were collected and identified as *Anopheles gambiae s.l*. in the two localities during both seasons using morphological identification. The distribution of the mosquito species between the two localities was similar (Bankeng = 975; Gounougou = 1010; *p* = 0.6), but in the rainy season, there were more mosquitoes compared to the dry season across both locations (dry season total = 425; rainy season total = 1560; *p* = 0.004) (Table S1).

To confirm the mosquito's species, molecular identification was conducted on all the 425 mosquitoes collected during the dry season, and 672 randomly selected mosquitoes from the rainy season collection. In Bankeng, during both seasons, the collection contained predominantly *An. gambiae ss* (*n* = 555, 99%), with only three *An. coluzzii* individuals. In Gounougou, during both seasons, the collection was comprised of predominantly *An. coluzzii* (*n* = 503, 93%) and the proportion *An. arabiensis* were only 7% (*n* = 36). In addition, this result showed the absence of *An. gambiae ss* in Gounougou. The microbiota diversity of two main *Anopheles* species (*An. gambiae* from Bankeng and *An. coluzzii* from Gounougou) was further characterized.

### Sample characteristics, 16S rRNA sequencing reads, and quality‐control statistics

Among the 120 samples sequenced, 118 have been selected because of a large number of high‐quality sequences obtained after quality control and were used for data analysis. A total of 4,833,081 paired‐end reads were generated. A total number of reads per sample (size per localities and season) is reported in Table S2. After quality filtering, 1,589,743 reads were assigned to ASVs at 99% identity and on average, there were 13,473 reads per mosquito sample (Table S2). To establish whether alpha diversity differs across mosquito species per season all samples were rarified to a depth of 1000 ASVs per sample, which was sufficient to capture the typical low microbiota diversity in individual mosquitoes (Figure S2).

### *Spatio‐temporal variation in microbial taxa associated with* Anopheles gambiae s.l.

**FIGURE 1:**
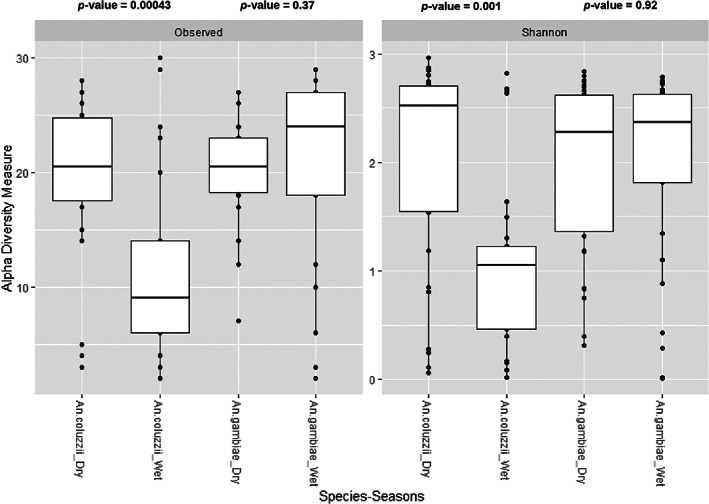
Shannon and observed diversity indices showed a significant difference in diversity of bacterial taxa between dry and wet seasons in *An. coluzzii* from Gounougou and in *An. gambiae* from Bankeng, there was no significant difference between dry and wet seasons. The comparison was performed using the Kruskal–Wallis test. Significance was determined at *p* < 0.05

In order to determine whether there was any spatio‐temporal variation in *Anopheles* microbiota, we compared microbial taxa and relative abundance between mosquitoes sampled in dry or wet seasons, from two different geographic localities. Since *An. gambiae ss* was only found in Bankeng and *An. coluzzii* in Gounougou, the data has been presented by species and localities stratified by seasons. For *An. gambiae ss* collected in Bankeng, no significant difference in Observed ASVs and Shannon indices of microbiota were noted between dry and wet seasons. However, for *An. coluzzii*, significant differences in Observed ASVs and Shannon index (Shannon index, *p* = 0.001; Observed ASVs, *p* = 0.00043) of microbiota were noted during the dry and wet seasons indicating that the dry season had the highest diversity than the wet season in Gounougou (Figure 1). In addition, a comparison of Observed ASVs and Shannon diversity indices showed a significant difference between *An. gambiae ss* collected at Bankeng and *An. coluzzii* collected at Gounougou with a higher microbial diversity in *An. gambiae ss* at Bankeng (Shannon index, *p* = 0.022; Observed ASVs, *p* = 0.0014) (Figure S3a,b).

**FIGURE 2:**
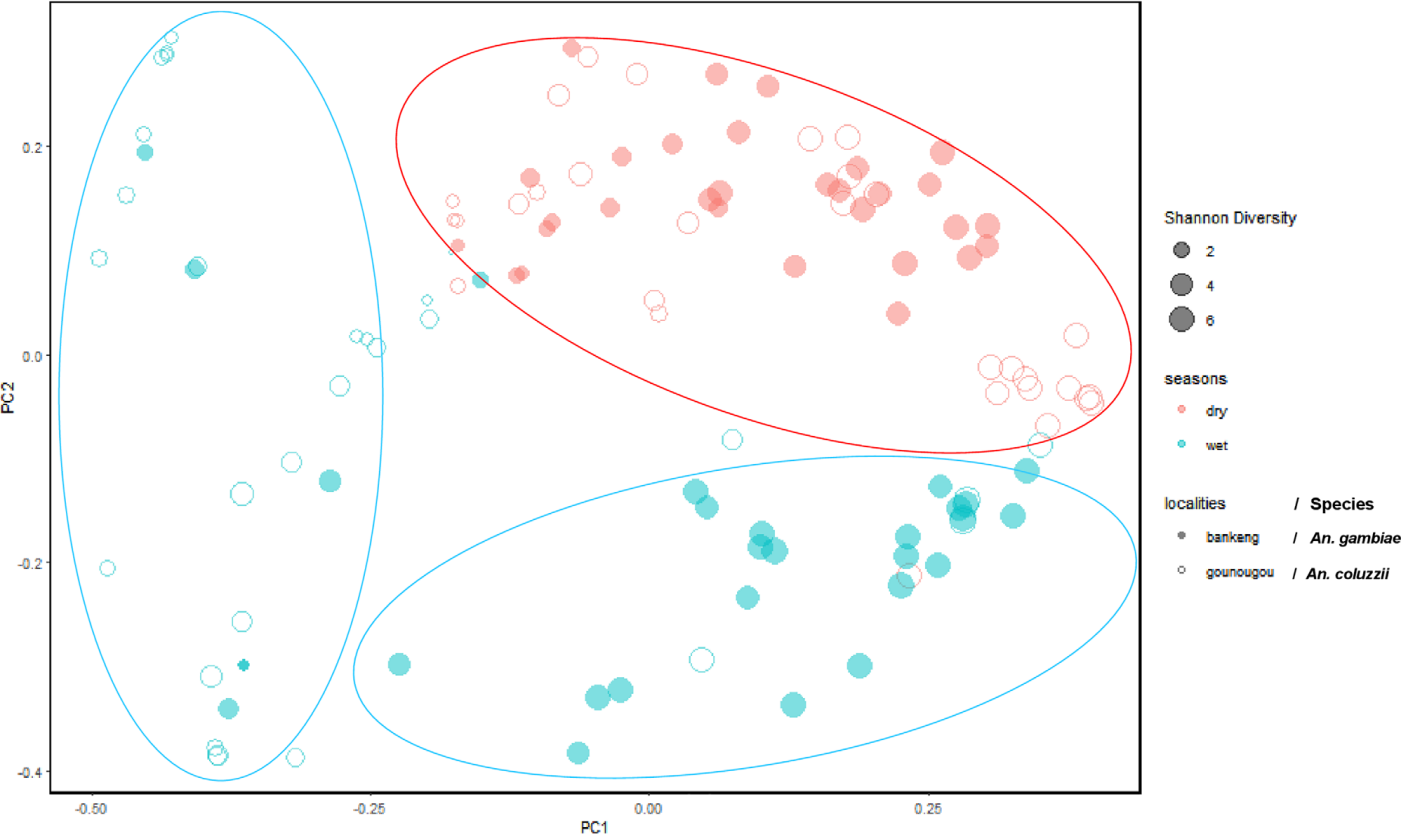
A principal coordinate analysis (PCoA) plot of bray‐Curtis distances between the microbiota of *Anopheles* collected from two different localities during the dry and wet season. Each point on the plot represents the microbial composition of a single mosquito. The bray‐Curtis comparison using permutational multivariate analysis of variance (999 permutations) showed a significant difference in microbial composition between samples collected between dry and wet seasons (Pseudo‐*F* = 10.45, *p* = 0.01). This is represented by the larger ovals or spheres where red represents both *Anopheles* species collected from Bankeng and Gounougou during the dry season and blue the mosquito's species collected from Bankeng and Gounougou during the wet season

Similarities and differences in the microbial composition between seasons and *Anopheles* species collected from different localities were examined. Bacterial communities varied significantly between seasons, with a clear separation of dry and wet season samples on the NMDS plot (Figure 2). NMDS based on the Bray‐Curtis distances was also used to observe that during the dry season the *An. gambiae* collected in Bankeng and *An*. *coluzzii* collected in Gounougou had a more similar bacterial community composition with higher proximity, while during the wet season, the two *Anopheles* species (*An. gambiae ss* and *An. coluzzii*) showed a significant difference in bacterial composition. Pairwise PERMANOVA comparisons of Bray‐Curtis distances also showed significant differences in microbial community structure of mosquitoes (*q* < 0.01) between every pair of seasons and every pair of species collected from different localities (Table S3). These results were corroborated by NMDS which showed that mosquito microbiota, clustered distinctly by seasons. We also investigated the similarity between the microbiota in both mosquito species collected from different localities during both seasons. For each species per locality tested, visualizations of Bray–Curtis diversity distance matrices showed the microbiota of mosquitoes collected in the dry season clustering distinctly away from those collected in the wet season (Figure 2 and Table S4).

**FIGURE 3:**
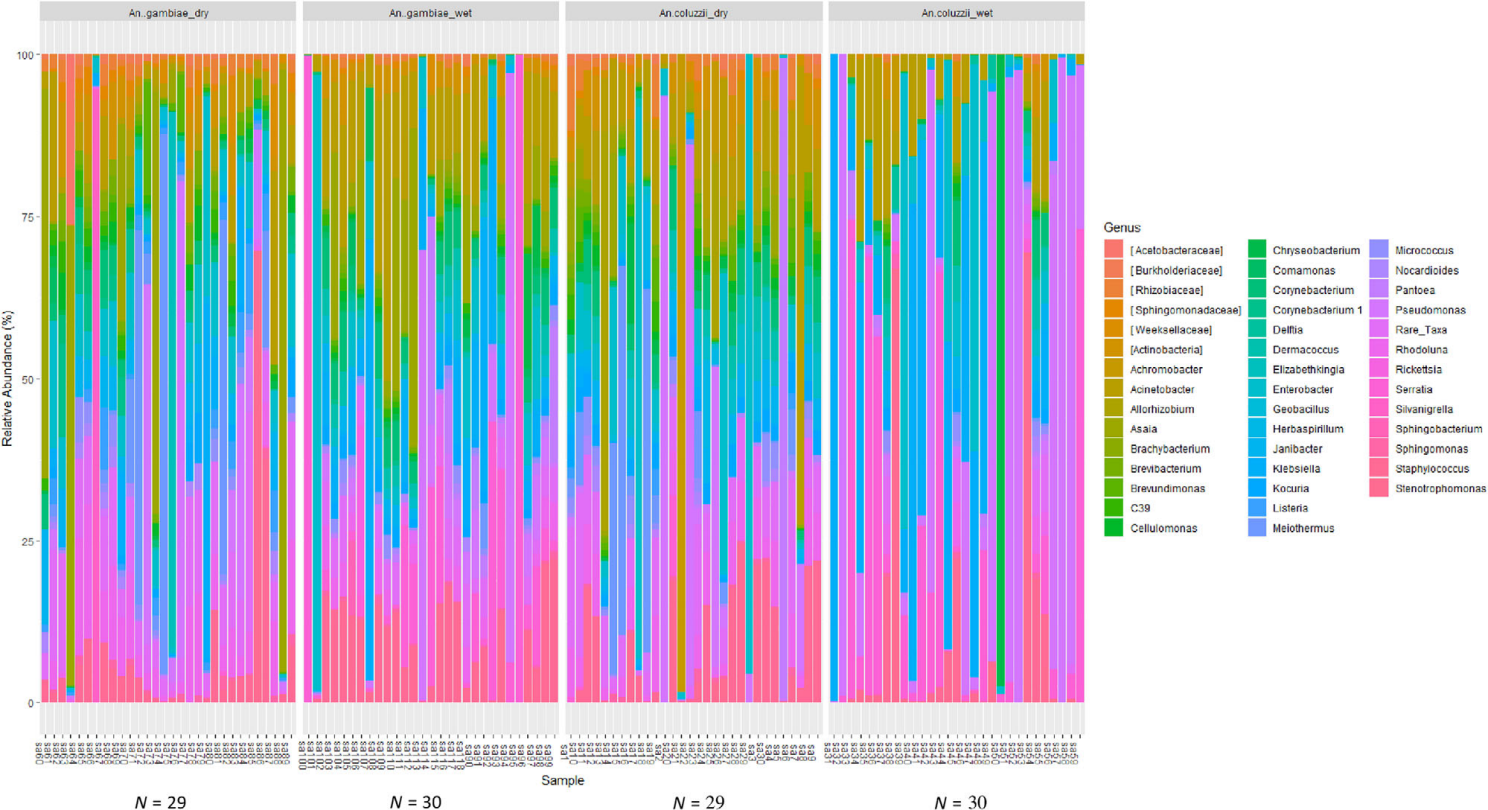
Bar plots showing the relative abundance of taxonomically annotated amplicon sequence variants (ASVs) from adults *An. gambiae ss* and *An. coluzzii*. ASVs showing an overall abundance equal to or greater than 0.1% were taxonomically annotated to the genus level. The bar plots show the relative abundance of annotated ASVs of individual mosquitoes across the two localities during both seasons. *N* represents the number of samples per group (*An. gambiae* collected in Bankeng during the dry and the wet seasons; *An. coluzzii* collected in Gounougou during the dry and the wet season). Microbiota was dominated by *Asaia* in Bankeng during the dry season, while the ASVs assigned to the genera *Serratia*, and *pseudomonas* were most predominant in the wet season. In Gounougou during the dry season, the ASVs assigned to the genera *Enterobacter* and *Pantoea* were most predominant, while the ASVs assigned to the genera *pseudomonas* and *Klebsiella* were most predominant in the wet season. ASVs that were not identified at the genus level are presented as unassigned taxa and rare taxa. Sa: sample.

The distribution of bacterial taxa among the 118 samples at the phylum, family and genus levels, and their relative abundance for individual samples in each sample group (two seasons and two localities) are reported. A total of 11 bacterial phyla, 66 families and 97 genera were identified across all samples (Table S5). Following taxonomic analysis of ASVs to the phylum level, 11 phyla were recovered, but only 5 of the 11 ASVs had an overall abundance equal to or greater than 0.1%. Most of the sequences were dominated by *Proteobacteria* (62.7%), followed by *Actinobacteria* (17.5%), *Firmicutes* (6.6%) *Deinococcus‐Thermus* (4.4%), and *Bacteroidetes* (3%) across all the samples (Figure S4). At the family level, 66 families were recovered, but only 29 of the 66 ASVs had an overall abundance equal to or greater than 0.1%. Many of the sequences were dominated by *Enterobacteriaceae* (26.3%), *Pseudomonadaceae* (13%), *Moraxellaceae* (8.3%), *Staphylococcaceae* (6%), and *Acetobacteraceae* (5.4%) across all the samples (Figure S5). At the genus level, 97 families were recovered, but only 42 of the 97 ASVs had an overall abundance equal to or greater than 0.1%. The most abundant genera were Pseudomonas (23.48%), *Klebsiella* (13.06%), *Enterobacter* (10.69%), *Serratia* (10.24%), *Acinetobacter* (5.96%), *Asaia* (4.82%), *Meiothermus* (3.54%) and *Pantoea* (3.20%) across all the samples (Figure 3 and Table S3). The mosquitoes' bacterial communities were different in composition and abundance among the seasons and locations of all mosquito species. The shifts in microbial compositions in predominant phyla, families and genera are shown in Table 1.

**TABLE 1:** Relative abundance (median in percentage and ranges) of all bacterial taxa significantly different identified in the dry and wet season for both localities

Localities	Bacterial	Dry_season	Wet_season	*p*‐value
Bankeng	*Phylum*			
	*Proteobacteria*	47.7 (6.3–99.1)	67.4 (28.8–100)	0.008
	*Deinococcus*	8.6 (0.0–83.2)	0.4 (0.0–6.9)	0.001
	*Family*			
	*Enterobacteriaceae*	8.6 (0.0–83.2)	8.6 (0.0–83.2)	0.001
	*Genus*			
	*Klebsiella*	3.6 (0.0–54.8)	9.6 (0.0–67.9)	0.0005
	*Serratia*	0.03 (0.0–0.44)	14.0 (0.0–99.8)	0.0001
	*Acinetobacter*	4.9 (0.0–29.9)	12.4 (0.0–44.3)	0.0006
	*Meiothermus*	8.6 (0.0–83.2)	0.37 (0.0–6.9)	0.0002
	*Pantoea*	0.0 (0.0–0.0)	2.97 (0.0–31.9)	0.0001
Gounougou	*Phylum*			
	*Proteobacteria*	52.8 (19.6–99.9)	86.18 (26.7–100)	0.002
	*Actinobacteria*	23.1 (0.0–52.4)	5.9 (0.0–15.6)	0.001
	*Deinococcus*	6.5 (0.0–56.8)	0.07 (0.0–0.49)	0.0002
	*Firmicutes*	8.6 (19.6–99.9)	2.7 (26.7–100)	0.0001
	*Family*			
	*Enterobacteriaceae*	37.7 (0.53–99.8)	54.0(0.58–99.2)	0.023
	*Pseudomonadaceae*	19.8 (0.0–93.4)	34.8 (0.08–99.4)	0.0003
	*Staphylococcaceae*	17.3 (0.00–24.4)	3.35 (0.00–22.9)	0.001
	*Genus*			
	*Klebsiella*	0.5 (0.0–2.9)	18.9 (0.0–99.8)	0.001
	*Enterobacter*	12.7 (0.5–95.5)	9.06 (0.0–70.4)	0.06
	*Serratia*	0.5 (0.0–12.5)	15.8 (0.0–73.9)	0.0001
	*Asaia*	1.9 (0.0–18.9)	0.00 (0.0–0.20)	0.0004
	*Meiothermus*	6.5 (0.0–56.9)	0.07 (0.0–49)	0.002
	*Pseudomonas*	8.7 (0.0–93.5)	28.6 (0.14–99.6)	0.003

**TABLE 2:** Differentially abundant ASVs identified by ANCOM when comparing samples from two seasons (dry vs. wet)

Significantly different taxa	W	Dry	Wet
*Listeria*	104	✔	
*Serratia*	102		✔
*Dermacoccus*	100		✔
*Pantoea*	91		✔
*Klebsiella*	82		✔
*Comamonas*	78		✔
*Eubacterium*	65		✔
*Meiothermus*	60	✔	
*Acinetobacter*	56		✔

*Note*: Taxa names into genus level based on corresponding taxonomy classification and seasons in which they were most abundant have been presented in this table.

Now visualizing the data for each sample in order to evaluate any inter‐individual variation at the genus level, the number of samples carrying the predominant bacteria were very different according to the season and *Anopheles* species per localities. Thus, the number of samples is presented for the eight predominant genera in Table S6. In summary, we can say that the bacterial composition in the individual sample from each group of samples was very different. For *An. gambiae* collected in Bankeng, during the dry season, *Enterobacter* and *Acinetobacter* were predominant respectively for 16 and 8 samples; while during the wet season, *Acinetobacter*, *Klebsiella*, *Serratia*, *Enterobacter*, *Asaia*, and *Pseudomonas* were predominant respectively for 21, 13, 12, 11,11, 8 samples. For *An. coluzzii* in Gounougou, during the dry season, *Enterobacter* and *Acinetobacter* were predominant respectively for 19 and 22 samples; while during the wet season, *Acinetobacter*, *Klebsiella*, *Serratia*, *Enterobacter*, *Pseudomonas* were predominant respectively for 12, 15, 11, 12,11, 11 samples (Table S6).

### Common or differentially abundant bacteria in mosquitoes and presence of main bacterial symbionts according to the seasons and mosquito species per localities

**FIGURE 4:**
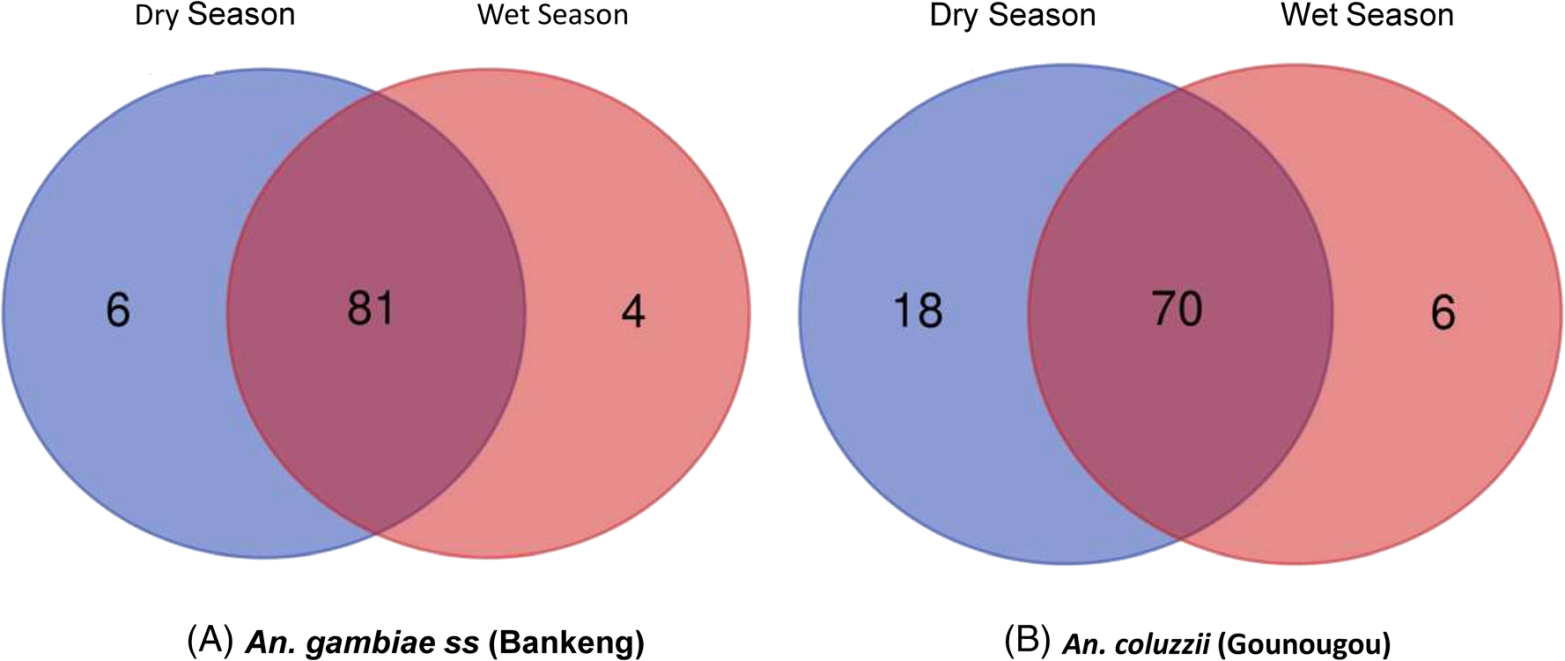
Venn diagrams showing the number of shared or unique bacterial ASVs among dry and wet seasons in each locality annotated to the genus level. (a) Number of unique and shared microbial taxa between dry and wet seasons in *An. gambiae ss* from Bankeng. (b) Number of unique and shared microbial taxa between dry and wet seasons in *An. coluzzii* from Gounougou

Some bacterial genera were specific to one group or shared between groups (*An. gambiae*‐Dry, *An. gambiae*‐Wet, *An. coluzzii*‐Dry and *An. coluzzii‐*Wet). In Bankeng (*An. gambiae*), 81 genera were shared between the dry and wet seasons, whereas in Gounougou (*An. coluzzii*) 70 genera were shared between the dry and wet seasons. Only 18 and 6 bacteria genera were unique to the dry season respectively to Gounougou and Bankeng (Figure 4a,b and Table S7). During the wet season, 7 and 4 bacterial genera were unique respectively to Gounougou and Bankeng. Across both seasons overall, 86 genera were shared between the dry and wet seasons and only 10 bacteria genera were unique to the dry season and only 2 bacteria genera were unique to the wet season (Figure S6A). Across both localities, 88 genera of bacteria were shared between the two *Anopheles* species and localities, and only 3 bacteria genera were observed in *An. gambiae ss* from Bankeng and only 6 bacteria genera were observed in *An. coluzzii* from Gounougou. (Figure S6B).

**FIGURE 5:**
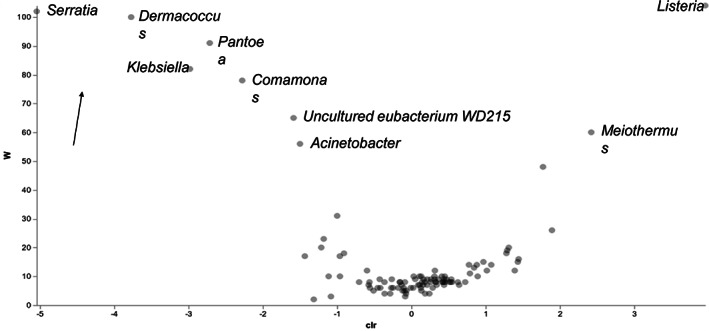
Volcano plots of differentially abundant bacterial taxa in *An. gambiae s.l* during the dry and wet seasons. The plots show the results of the analysis of the composition of microbiomes (ANCOM) tests for differentially abundant microbial taxa between dry and wet seasons, with an effect size set to log F ≥ 20, and a cut‐off of differential abundance set to W ≥ 20 (i.e., a taxon was differentially abundant across seasons if the ratio of its abundance to those of at least 20 other taxa (25% of all included taxa) differed significantly across seasons). Truly different taxa are depicted as one moves towards the far right (high W‐statistic) as indicated by the arrow on the figure. Differentially abundant taxa are highlighted (blue shaded area) and the taxa names and seasons in which they were most abundant are presented in Table 2

Overall, two ASVs were more abundant in the dry season (*Listeria* and *Meiothermus*), while seven were more abundant in the wet season (*Serratia*, *Dermacoccus*, *Pantoea*, *Klebsiella*, *Comamonas*, *Eubacterium*, *Acinetobacter*) (Figure 5). In both *Anopheles* species and localities, five ASVs were found to be differentially more abundant in Bankeng (*An. gambiae*) compared to Gounougou (*An. coluzzii*), with one more abundant (*Listeria*) in Bankeng, and four *Rhizobium*, *Dermacoccus*, an *uncultured soil bacterium* and *Corynebacterium1* more abundant in Gounougou. Among significant differential bacteria observed, *Listeria* was only detected in the dry season and only in *An. gambiae ss* from Bankeng, while an *uncultured soil bacterium* was only detected in *An. coluzzii* from Gounougou (Figure S7 and Table S8).

**FIGURE 6:**
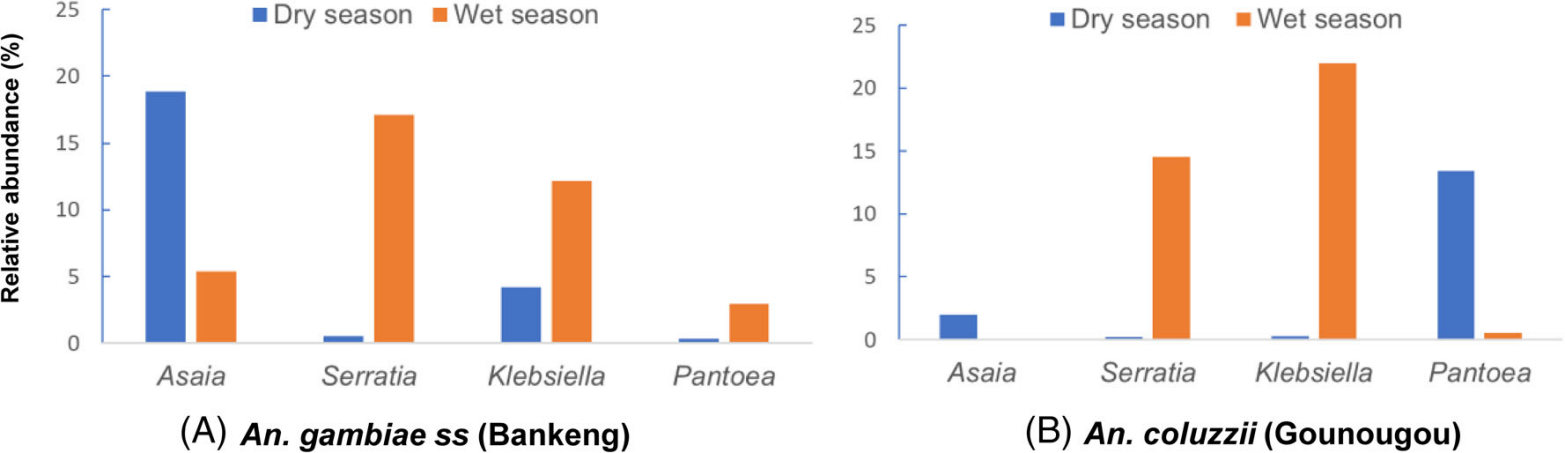
Bar plots showing the relative abundance of taxonomically annotated amplicon sequence variants (ASVs) of different bacterial symbionts according to the dry and wet seasons in each locality and mosquitoes' species. A is represented by the relative abundance of mains bacterial symbionts for *An. gambiae ss* collected in Bankeng and B is represented by the relative abundance of mains bacterial symbionts for *An*. *coluzzii* collected in Gounougou

Furthermore, special attention was paid to bacterial symbionts, due to their role in mosquito physiology and their interaction with *Plasmodium*. A total of four major bacterial symbionts (*Asaia*, *Serratia*, *Klebsiella*, and *Pantoea*) were detected in both seasons and mosquito's species from both localities. We found that the relative abundance of these bacterial symbionts was very different according to the seasons and *Anopheles* species per localities. For example, in Bankeng where we only have *An. gambiae ss*, *Asaia* (19.5%) was most predominant in the dry season compared to the wet season, while *Serratia* (16.8%), and *Klebsiella* (12.1%) were most predominant in the wet season (Figure 6a, Table S9). In Gounougou where we only have *An. coluzzii*, *Pantoea* (13.4%) was most predominant in the dry season, while *Serratia* (14.5%) and *Klebsiella* (21.9%) were most predominant in the wet season (Figure 6b, Table S6). Combining seasons and *Anopheles* species collected from different localities, *Asaia* is most predominant during the dry season in *An. gambiae ss*; *Pantoea* is most predominant during the dry season in *An. coluzzii*; *Serratia* and *Klebsiella* are most predominant during the wet season in both species.

## DISCUSSION

The mosquito microbial composition is complex and depends on several factors such as the acquisition of environmental microbes (localities) and seasonality. Here, we characterized the bacterial composition in *An. coluzzii* and *An. gambiae* sampled from two different eco‐geographical localities during the dry and the wet seasons. Overall, our data suggest that the bacterial communities of the two *Anopheles* species vary significantly between the seasons. This corroborates previous studies showing that seasonality impacts the adult mosquito microbiota composition (Akorli et al., 2016).

We report the diversity of the microbiota composition of two *Anopheles* species collected during the dry and wet seasons. These were *An. gambiae* and *An. coluzzii* collected from two localities in Cameroon including the Sahelian area (Gounougou) and the forested area (Bankeng). *An. gambiae* was the dominant species collected in Bankeng and *An. coluzzii* was the dominant species collected in Gounougou. Previous observations in the southern forested areas and North areas of Cameroon have demonstrated the predominance of *An. gambiae ss* in the forest area and *An. coluzzii* in the Sudano‐Sahelian areas (Antonio‐Nkondjio et al., 2008; Bigoga et al., 2007; Elanga‐Ndille et al., 2020; Fadel et al., 2019). There was a significant difference in the composition of mosquito microbiota among mosquito species indicating the great inter‐sample variation in the bacterial taxa present with the dominance of some bacterial ASVs.

The bacterial composition in mosquitoes was dominated by five main phyla, 29 families and 42 genera across all samples. Among the five bacterial phyla include proteobacteria, a highly diverse phylum that contains a variety of bacterial species that are adapted to a wide range of environments. Its dominance in mosquito midguts has already been reported in Vietman and in Senegal, in *Anopheles* mosquitoes (Bassene et al., 2018; Ngo et al., 2016). These bacteria phyla are also commonly reported in other mosquitoes and insects (Osei‐Poku et al., 2012; Wang et al., 2011). However, the comparison of bacterial composition at the phylum level by season showed a higher abundance of *Proteobacteria* in mosquitoes collected in the wet compared to dry seasons, whereas other phyla were predominant in the dry season. The previous results have demonstrated a higher microbiota diversity in the dry season collections compared to wet, in Ghanian populations of *Anopheles* (Akorli et al., 2016).

At the genus level, *Asaia*, *Serratia*, *Enterobacter*, and *Pseudomonas* were predominant across all the samples. *Asaia* was the most dominant genus in Bankeng (*An. gambiae*) during the dry season as previously observed in *Anopheles* adults collected in the field in Burkina‐Faso (Damiani et al., 2010), while *Serratia* was the most dominant in the wet season. Similarly, *Enterobacter* was most dominant in Gounougou (*An. coluzzii*), while the ASVs assigned to the genera *Pseudomonas* was most dominant in the wet season. Previous studies report the predominance of *Serratia* and *Enterobacter* in *Anopheles* adults collected in the field (Dennison et al., 2014; Yadav et al., 2015); *Pseudomonas* has already been identified in *An. gambiae s.l*. collected in Mali (Diallo et al., 2015). These variations of bacteria according to the localities and seasons could be explained by the variability in physiological conditions of individual mosquitoes or habitat environmental conditions, and genetic factors (Osei‐Poku et al., 2012). These variations may indicate the population and species‐level variation in vector competence in different localities. Certain bacterial species can increase (Apte‐Deshpande et al., 2012) or reduce (Ramirez et al., 2012) vector susceptibility to pathogens and have the potential to alter vectorial capacity (Cansado‐Utrilla et al., 2021). In addition, the presence of various bacteria in the Cameroonian vector species during different seasons would suggest a facilitated dispersion among the mosquito communities; implying that they will be more susceptible to incorporating the bacteria after reintroduction.

There were differences in bacterial diversity and evenness between the mosquito species and the seasons studied. The Shannon and Observed indices show that the microbiota of *An. gambiae* from Bankeng was significantly diverse and distributed compared to *An. coluzzii* from Gounougou. This significant difference between mosquito species across different localities suggests that the sampling site environment is a key determinant of the bacterial profiles in mosquitoes. This could be because breeding sites where most of the mosquitoes acquired bacteria were different between both localities because *An. gambiae* has been collected only in Bankeng and *An. coluzzii* has been collected only in Gounougou. Similar results were reported in *Anopheles gambiae* in Mali and in Ghana regarding the microbiota in different localities (Akorli et al., 2016; Krajacich et al., 2017). In addition, Shannon and Observed diversity indices between the dry and wet seasons in each locality showed significant differences in microbiota only in Gounougou for *An. coluzzii*, where microbiota diversity was higher in the dry season compared to the wet season. In the rainy season, the number of breeding sites increased due to the presence of both cotton farming and rice cultivation which are known as suitable breeding sites for this species. However, Shannon and Observed diversity indices between dry and wet seasons were similar in Bankeng, suggesting that the mosquito plays an important role in regulating the colonization and assembly of bacterial communities.

The beta diversity results showed a significant difference between seasons (dry vs. wet) and *Anopheles* species collected from different localities (Bankeng vs. Gounougou). The Bray‐Curtis distances analysis presented here showed a clear separation on the beta diversity plot, between dry and wet seasons. This largely supports the effect of seasons on bacterial composition, which is also consistent with previous studies (Akorli et al., 2016; Krajacich et al., 2017). This variation in some groups of mosquitoes could result from the presence or absence of certain taxa, as well as some dominant genera. This could be also explained by the fact that the rice farms could be the only main breeding site for larvae mosquitoes. Also, our result showed a significant difference in the composition of the microbiota in *An. gambiae* and *An. coluzzii* in the wet season. A similar pattern of β‐diversity variation, driven by the less‐frequent microbes, was observed in other insects, including the *pine weevil*, *bark beetles*, *brown planthopper* and *Haemaphysalis longicornis* (Berasategui et al., 2016; Durand et al., 2015; Zhang, Huang, et al., 2019; Zhang, Yu, et al., 2019). In contrast, there was no separation between the microbiota of *Anopheles* mosquitoes collected in both localities during the dry season. This could be explained by the same permanent breeding site (rice cultivation) during this season.

We also identified some bacteria symbionts with a high number of reads in our microbiota data. One promising mosquito‐borne disease control approach would be the use of symbiotic bacteria naturally associated with mosquitoes, to impede the development of *Plasmodium* during the sporogonic cycle inside the mosquito (Mancini et al., 2016). This included four main bacterial symbionts (*Asaia*, *Serratia*, *Klebsiella*, and *Pantoea*) with variable relative abundance by *Anopheles* species and seasons (Figure 6). These variations could be explained by the fact that breeding sites were very variable between both localities and seasons, resulting in variable bacterial symbionts. The capacity to colonize and remain among the bacterial communities independent of *Anopheles* species and geographical localities will guarantee their success in a paratransgenesis approach. Some members of these bacterial taxa are known to be effective against *Plasmodium* development (Bando et al., 2013; Cirimotich et al., 2011), while others have been reported to be positively associated with *P. falciparum* infections in mosquitoes (Boissière et al., 2012). *Asaia*, *Serratia*, *Pantoea*, *Klebsiella* have been proposed as promising symbiotic control agents of malaria vectors such as *An. gambiae* by paratrangenesis. Bacteria from the genus *Asaia* have been evaluated as paratransgenic agents to control malaria transmission (Damiani et al., 2010; Favia et al., 2007; Maffo et al., 2021). Bacteria from this genus are considered viable candidates due to their stable association with anopheline mosquitoes throughout the body and different life stages; they can also be easily cultivated and transformed. A previous study on genetically modified *Serratia* shows promise for controlling malaria by killing *Plasmodium* ookinete/oocysts in the mosquito midgut, the ‘location’ in which the cycle within the mosquito undergoes a severe bottleneck (Wang et al., 2011). The natural mosquito symbiont *Pantoea* can cross‐colonize several mosquito species and is readily transformed and cultured; thus, *Pantoea* has been proposed for paratransgenic applications (Bisi & Lampe, 2011; Djadid et al., 2011). Finally, in *An. stephensi* the presence of *Klebsiella* reduced the sporogonic development of *P. berghei* (Jadin, 1967). Future studies should be directed to characterize these bacteria symbionts *Asaia*, *Serratia*, *Klebsiella*, *Pantoea*, to evaluate their biological functions in the *Anopheles gambiae* mosquitoes and potential antiparasitic activity in the Cameroonian context.

## CONCLUSION

This study has shown for the first‐time significant differences in microbiota composition between dry and wet seasons in two *Anopheles* species collected from two different localities in Cameroon. We provide evidence that the seasons were significantly affecting both the bacterial composition and relative abundance of the bacterial genera with more microbial diversity in the dry season in both species collected from two different localities (Gounougou and Bankeng). In addition, this study provides evidence of the presence of potential bacteria symbionts that can be used to develop novel approaches for mosquito control. Further studies will evaluate the suitability of these bacteria symbionts as candidates for paratransgenesis.

## AUTHOR CONTRIBUTIONS

Maurice Marcel Sandeu and Charles S. Wondji conceived and designed the study. Maurice Marcel Sandeu and Claudine Grâce Tatsinkou Maffo collected the samples on the field. Maurice Marcel Sandeu and Claudine Grâce Tatsinkou Maffo performed the experimental analysis. Maurice Marcel Sandeu and Nsa Dada performed the data analysis. Maurice Marcel Sandeu wrote the manuscript with contributions from Nsa Dada, Grant L. Hughes, Flobert Njiokou and Charles S. Wondji.

## CONFLICT OF INTEREST

The authors declare that they have no competing interests.

## Supporting information


**Figure S1.** Map of Cameroon showing Bankeng and Gounougou localities, where the adult mosquito was collected for studying the microbiota diversity. Adult female *Anopheles gambiae s.s*. were collected from Bankeng village and Adult female *Anopheles coluzzii* were collected from Gounougou village.

**Figure S2.** Rarefaction curves and depth. The following figures show rarefaction curves of the alpha diversity index (Observed ASVs and Shannon) for each mosquito tested. All curves plateaued, indicating that additional sampling efforts did not result in changes in abundance and evenness of microbial taxa per sample. At each sampling depth shown, each curve shows the average Observed and Shannon diversity value, along with the range (boxplots‐minimum, median and maximum) of values from 10 rarefaction iterations.

**Figure S3.** (a) Alpha diversity comparisons between *An. gambiae* and *An. coluzzii* using Observed and Shannon diversity indices. The results showed that the species (*An. gambiae* and *An. coluzzii*) affect mosquito microbiota diversity (Shannon index, *p* = 0.022; Observed ASVs, *p* = 0.0014). Comparisons were performed using the Kruskal–Wallis pairwise tests. (b) Alpha diversity comparisons between Bankeng and Gounougou using Observed and Shannon diversity indices. The results showed that the localities (Bankeng and Gounougou) affect mosquito microbiota diversity (Shannon index, *p* = 0.022; Observed ASVs, *p* = 0.0014). Comparisons were performed using the Kruskal–Wallis pairwise tests.

**Figure S4.** Bar plots showing the relative abundance of taxonomically annotated amplicon sequence variants (ASVs) from adults *An. gambiae ss* and *An. coluzzii*. ASVs showing an overall abundance equal to or greater than 0.1% were taxonomically annotated to the phylum level. The bar plots show the relative abundance of annotated ASVs for individual samples across the two localities during both seasons. *N* represents the number of samples per group (*Anopheles gambiae* collected in Bankeng during the dry and the wet seasons; *Anopheles coluzzii* collected in Gounougou during the dry and the wet season). In general, the microbiota was dominated by *Proteaobacteria* followed by *Actinobacteria*, *Firmicutes* Deinococcus*‐Thermus*, and *Bacteroidetes*.
Footnote. sa: sample

**Figure S5.** Bar plots showing the relative abundance of taxonomically annotated amplicon sequence variants (ASVs) from adults *An. gambiae ss* and *An. coluzzii*. ASVs showing an overall abundance equal to or greater than 0.1% were taxonomically annotated to the family level. The bar plots show the relative abundance of annotated ASVs of individual mosquitoes across the two localities during both seasons. N represents the number of samples per group (*Anopheles gambiae* collected in Bankeng during the dry and the wet seasons; *Anopheles coluzzii* collected in Gounougou during the dry and the wet season). The microbiota was dominated by *Acetobacteraceae* in Bankeng during the dry season, while the ASVs assigned to *Enterobacteriaceae* were most predominant during the wet season. In Gounougou during the dry season the ASVs assigned to the family *Acetobacteraceae*, *Moraxellaceae and Staphylococcaceae* were most predominant, while the ASVs assigned to the *Enterobacteriaceae* and *Pseudomonadaceae* were most predominant in the wet season. ASVs that were not identified at the genus level are presented as unassigned taxa and rare taxa.
Footnote. sa: sample

**Figure S6.** Venn diagrams showing the number of shared or unique bacterial ASVs among dry and wet seasons and among localities annotated to the genus level. (A) Number of unique and shared microbial taxa between seasonal (dry vs. wet). (B) Number of unique and shared microbial taxa between collection areas or *Anopheles* species (Bankeng vs. Gounougou).

**Figure S7.** Volcano plots of differentially abundant bacterial taxa in *An. gambiae* between the two localities. The plots show the results of the analysis of the composition of microbiomes (ANCOM) tests for differentially abundant microbial taxa between Bankeng and Gounougou, with an effect size set to log F ≥ 20, and a cut‐off of differential abundance set to W ≥ 20 (i.e., a taxon was differentially abundant across localities if the ratio of its abundance to those of at least 20 other taxa [25% of all included taxa] differed significantly across seasons). Truly different taxa are depicted as one moves towards the far right (high W‐statistic) as indicated by the arrow on the figure. Differentially abundant taxa are highlighted (blue shaded area) and the taxa names and locations in which they were most abundant are presented in Table S8.

**Table S1.** Number of *Anopheles sl* mosquitoes collected according to the localities and seasons
**Table S2.** Sequencing outputs and proportion of reads used for downstream analysis following quality control and dereplication
**Table S3.** Beta‐diversity comparisons show differential bacterial composition between the localities and between seasons: Pair‐wise comparisons of beta diversity (Bray Curtis) of mosquitoes' microbiota between seasons (dry vs. wet), and between localities (Bankeng vs. Gounougou) showed significant differences in bacterial composition. Comparisons were conducted using PERMANOVA (999 permutations) tests with Benjamini‐Hochberg FDR correction (*q*‐value). Significance is set to *q*‐value (adjusted *p*‐value).
**Table S4.** Beta‐diversity comparisons show differential bacterial composition between seasons in each locality. Pair‐wise comparisons of beta diversity (Bray Curtis) of mosquito's microbiota between seasons in each locality (Bankeng vs. Gounougou) showed significant differences in bacterial composition. Comparisons were conducted using PERMANOVA (999 permutations) tests with Benjamini‐Hochberg FDR correction (*q*‐value). Significance is set to *q*‐value (adjusted *p*‐value).
**Table S5.** Relative abundance of each bacterium at the Phylum, family, and genus level for each mosquito samples according to the localities and seasons
**Table S6.** Number of individual samples with a predominant bacterial at the genus level
**Table S7.** Summary of taxonomic annotation and frequency of ASVs used to construct the Venn diagram
**Table S8.** Differentially abundant ASVs identified by ANCOM when comparing samples from two localities (Bankeng vs. Gounougou). Taxa names into genus level based on corresponding taxonomy classification and localities in which they were most abundant have been presented in this table.
**Table S9.** Relative abundance of main bacteria symbionts according to the localities and seasons.

## Data Availability

The data that support the findings of this study are available from the corresponding author upon reasonable request.
