# Supplementary figures and images for: Seasonal variation of microbiota composition in Anopheles gambiae and Anopheles coluzzii in two different eco‐geographical localities in Cameroon
Source: Med Vet Entomol. 2022 May 17;36(3):269–82. doi: 10.1111/mve.12583 (PMC10286663; doi:10.1111/mve.12583)

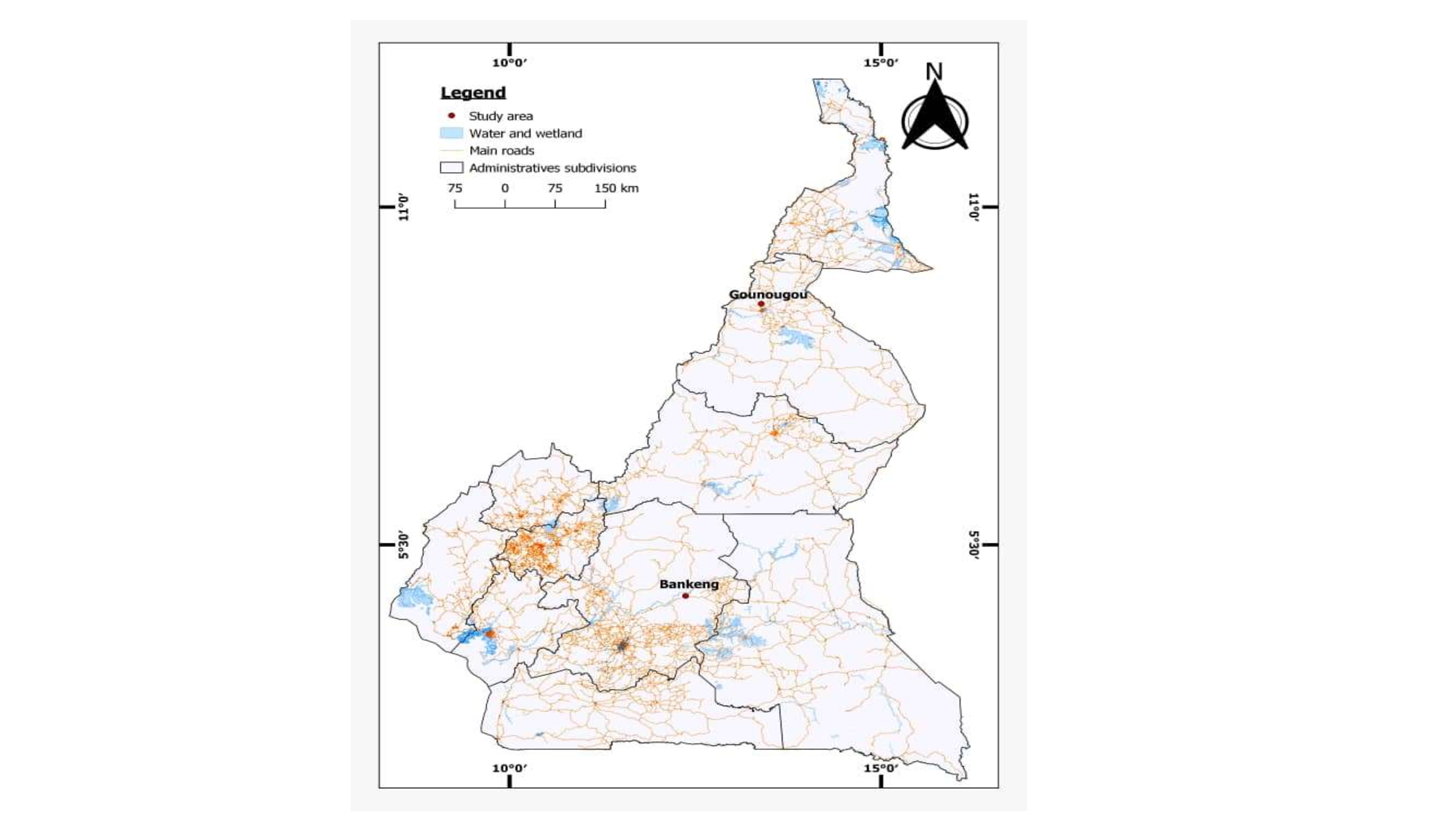

Supplement: Supplementary file 1 — Figure S1. Map of Cameroon showing Bankeng and Gounougou localities, where the adult mosquito was collected for studying the microbiota diversity. Adult female Anopheles gambiae s.s. were collected from Bankeng village and Adult female Anopheles coluzzii were collected from Gounougou village. [file MVE-36-269-s003.tiff]

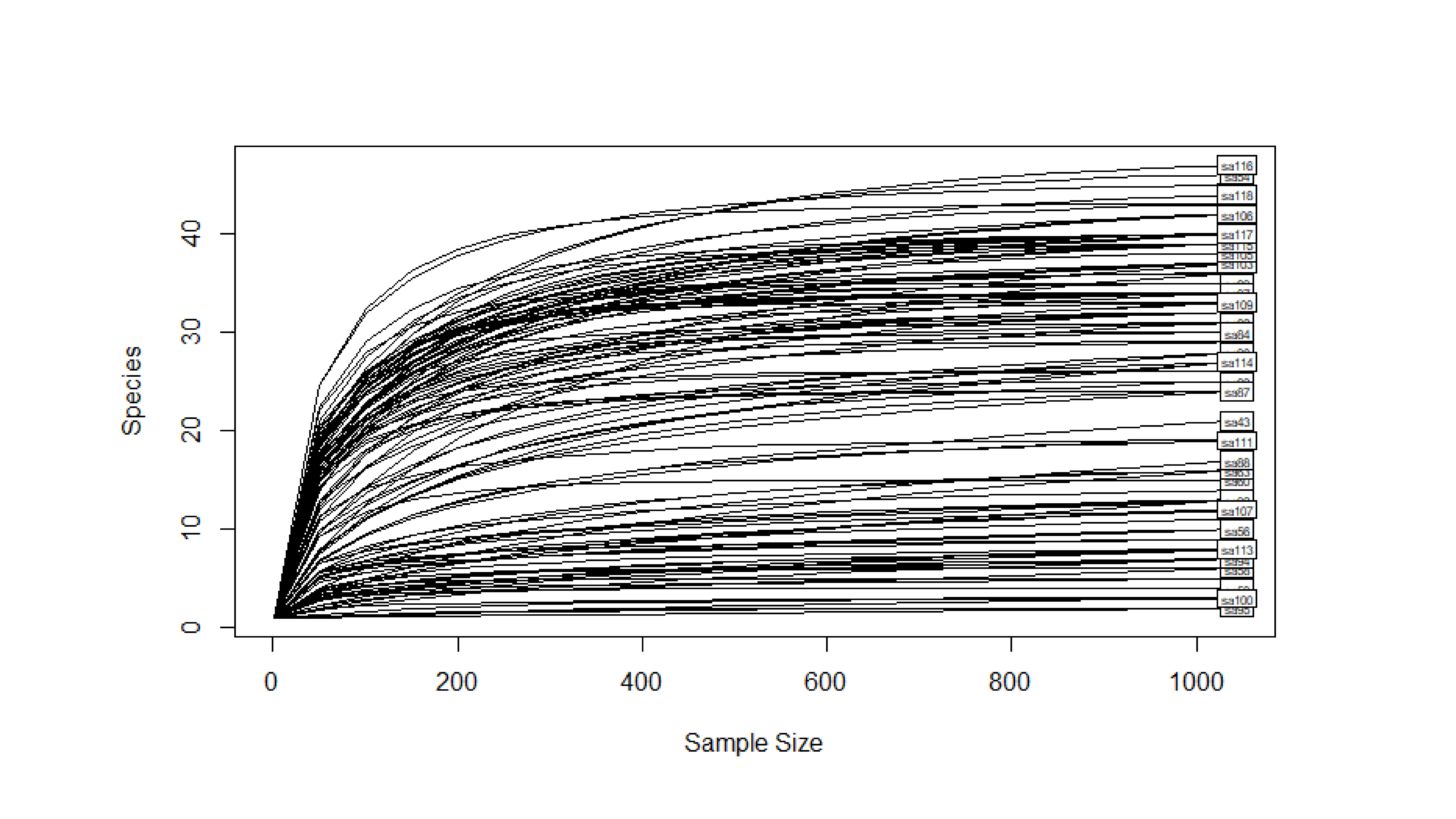

Supplement: Supplementary file 2 — Figure S2. Rarefaction curves and depth. The following figures show rarefaction curves of the alpha diversity index (Observed ASVs and Shannon) for each mosquito tested. All curves plateaued, indicating that additional sampling efforts did not result in changes in abundance and evenness of microbial taxa per sample. At each sampling depth shown, each curve shows the average Observed and Shannon diversity value, along with the range (boxplots‐minimum, median and maximum) of values from 10 rarefaction iterations. [file MVE-36-269-s004.tiff]

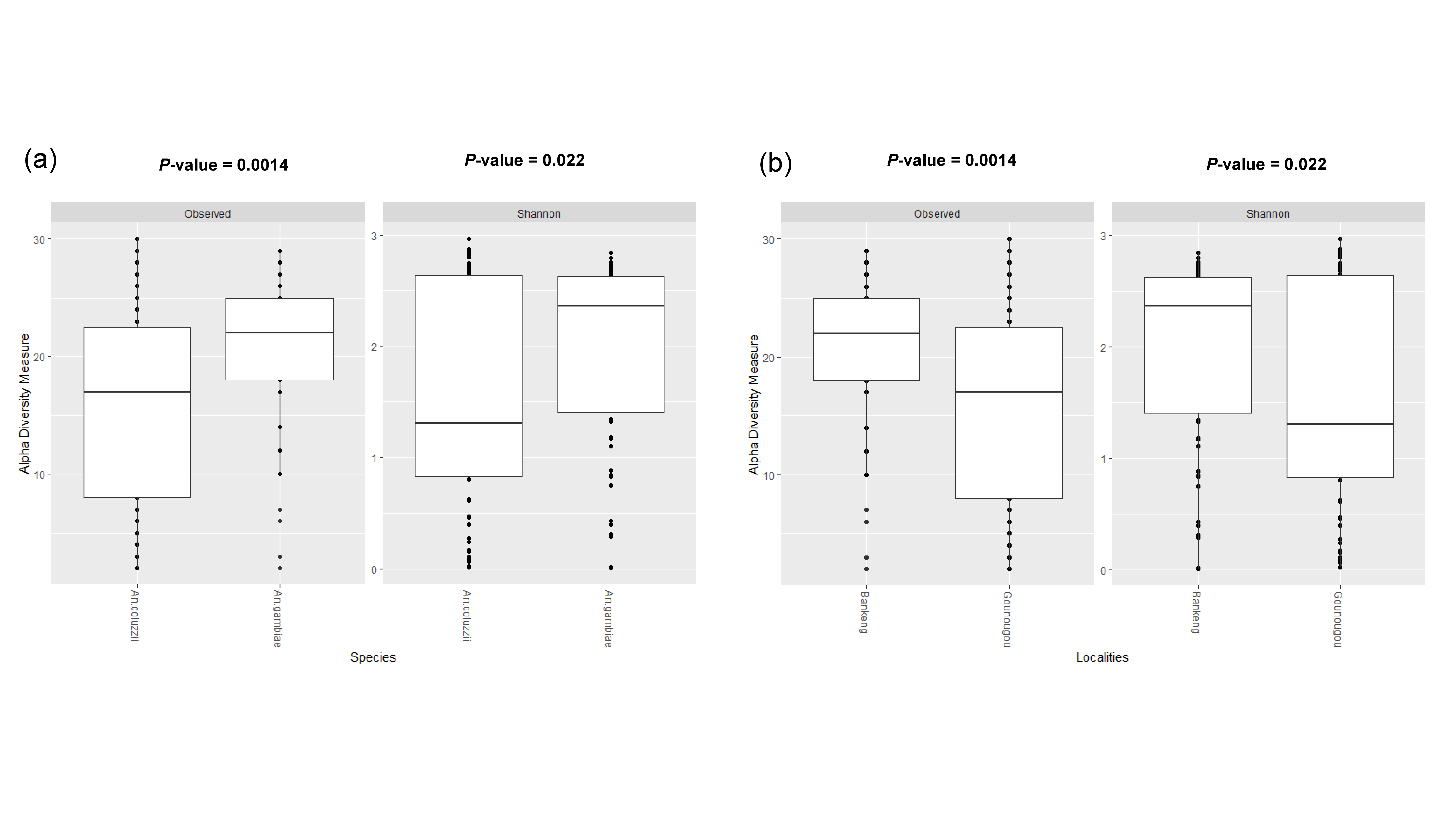

Supplement: Supplementary file 3 — Figure S3. (a) Alpha diversity comparisons between An. gambiae and An. coluzzii using Observed and Shannon diversity indices. The results showed that the species (An. gambiae and An. coluzzii) affect mosquito microbiota diversity (Shannon index, p = 0.022; Observed ASVs, p = 0.0014). Comparisons were performed using the Kruskal–Wallis pairwise tests. (b) Alpha diversity comparisons between Bankeng and Gounougou using Observed and Shannon diversity indices. The results showed that the localities (Bankeng and Gounougou) affect mosquito microbiota diversity (Shannon index, p = 0.022; Observed ASVs, p = 0.0014). Comparisons were performed using the Kruskal–Wallis pairwise tests. [file MVE-36-269-s001.tiff]

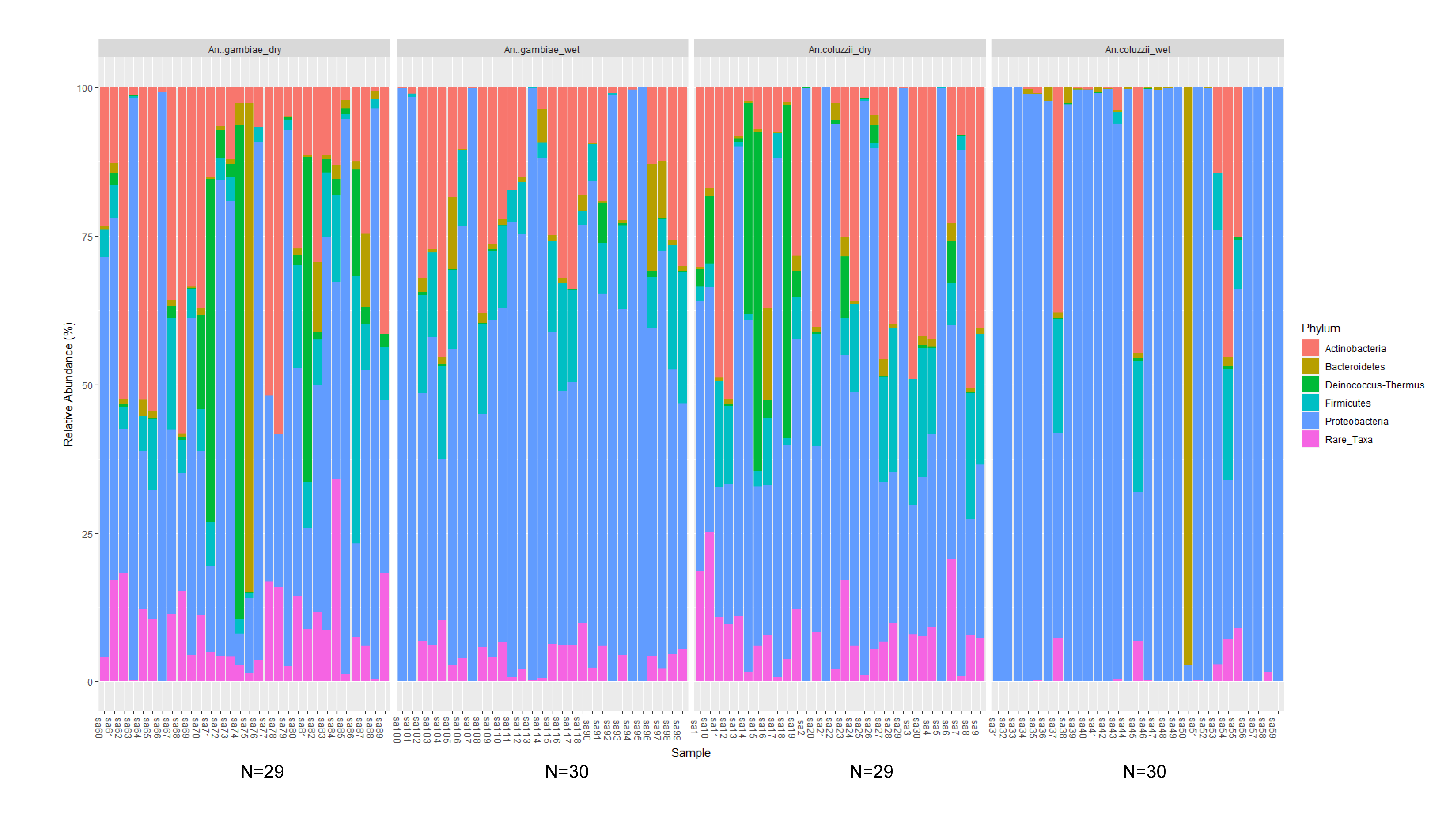

Supplement: Supplementary file 4 — Figure S4. Bar plots showing the relative abundance of taxonomically annotated amplicon sequence variants (ASVs) from adults An. gambiae ss and An. coluzzii. ASVs showing an overall abundance equal to or greater than 0.1% were taxonomically annotated to the phylum level. The bar plots show the relative abundance of annotated ASVs for individual samples across the two localities during both seasons. N represents the number of samples per group (Anopheles gambiae collected in Bankeng during the dry and the wet seasons; Anopheles coluzzii collected in Gounougou during the dry and the wet season). In general, the microbiota was dominated by Proteaobacteria followed by Actinobacteria, Firmicutes Deinococcus‐Thermus, and Bacteroidetes. Footnote. sa: sample [file MVE-36-269-s002.tiff]

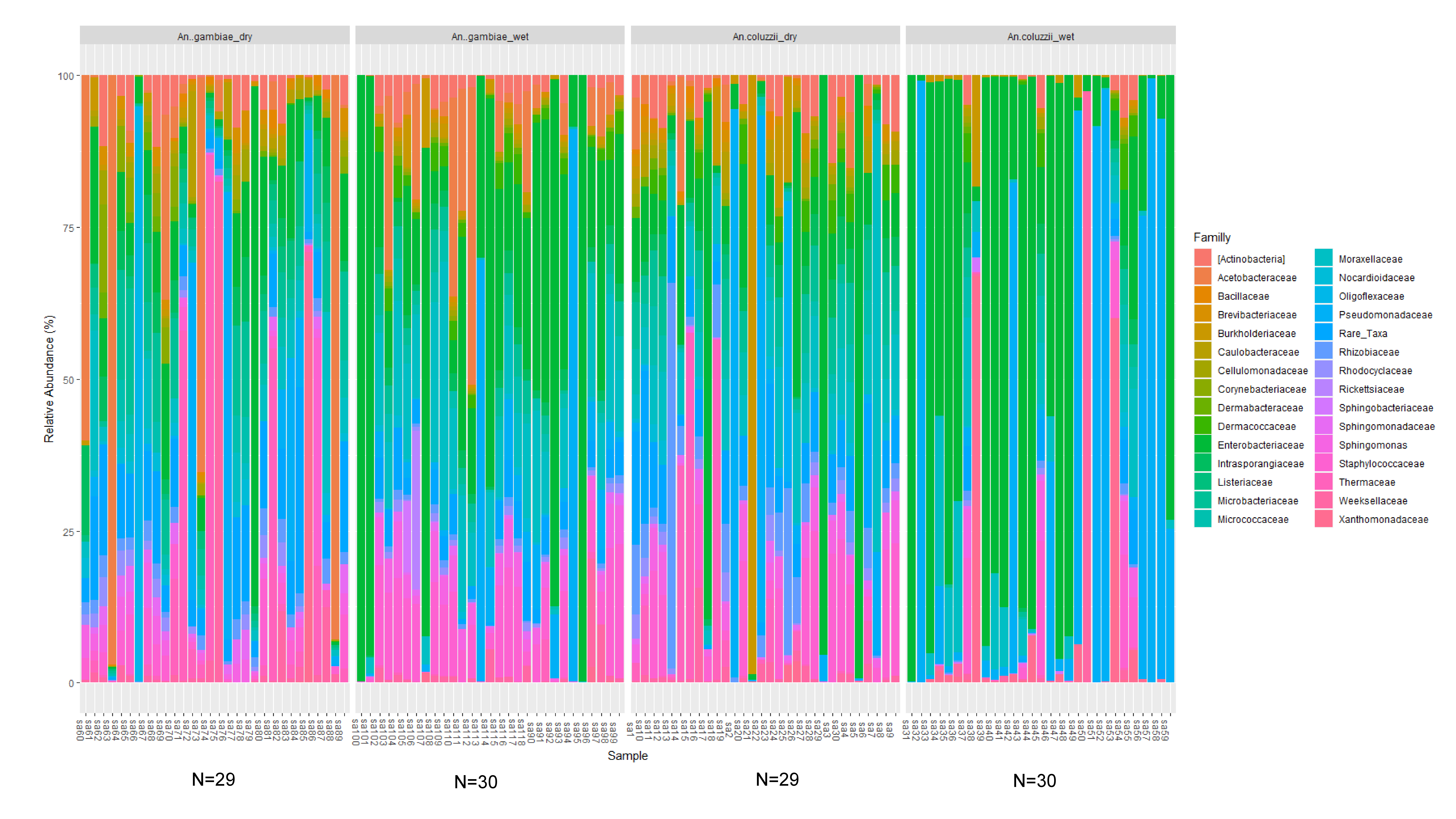

Supplement: Supplementary file 5 — Figure S5. Bar plots showing the relative abundance of taxonomically annotated amplicon sequence variants (ASVs) from adults An. gambiae ss and An. coluzzii. ASVs showing an overall abundance equal to or greater than 0.1% were taxonomically annotated to the family level. The bar plots show the relative abundance of annotated ASVs of individual mosquitoes across the two localities during both seasons. N represents the number of samples per group (Anopheles gambiae collected in Bankeng during the dry and the wet seasons; Anopheles coluzzii collected in Gounougou during the dry and the wet season). The microbiota was dominated by Acetobacteraceae in Bankeng during the dry season, while the ASVs assigned to Enterobacteriaceae were most predominant during the wet season. In Gounougou during the dry season the ASVs assigned to the family Acetobacteraceae, Moraxellaceae and Staphylococcaceae were most predominant, while the ASVs assigned to the Enterobacteriaceae and Pseudomonadaceae were most predominant in the wet season. ASVs that were not identified at the genus level are presented as unassigned taxa and rare taxa. Footnote. sa: sample [file MVE-36-269-s006.tiff]

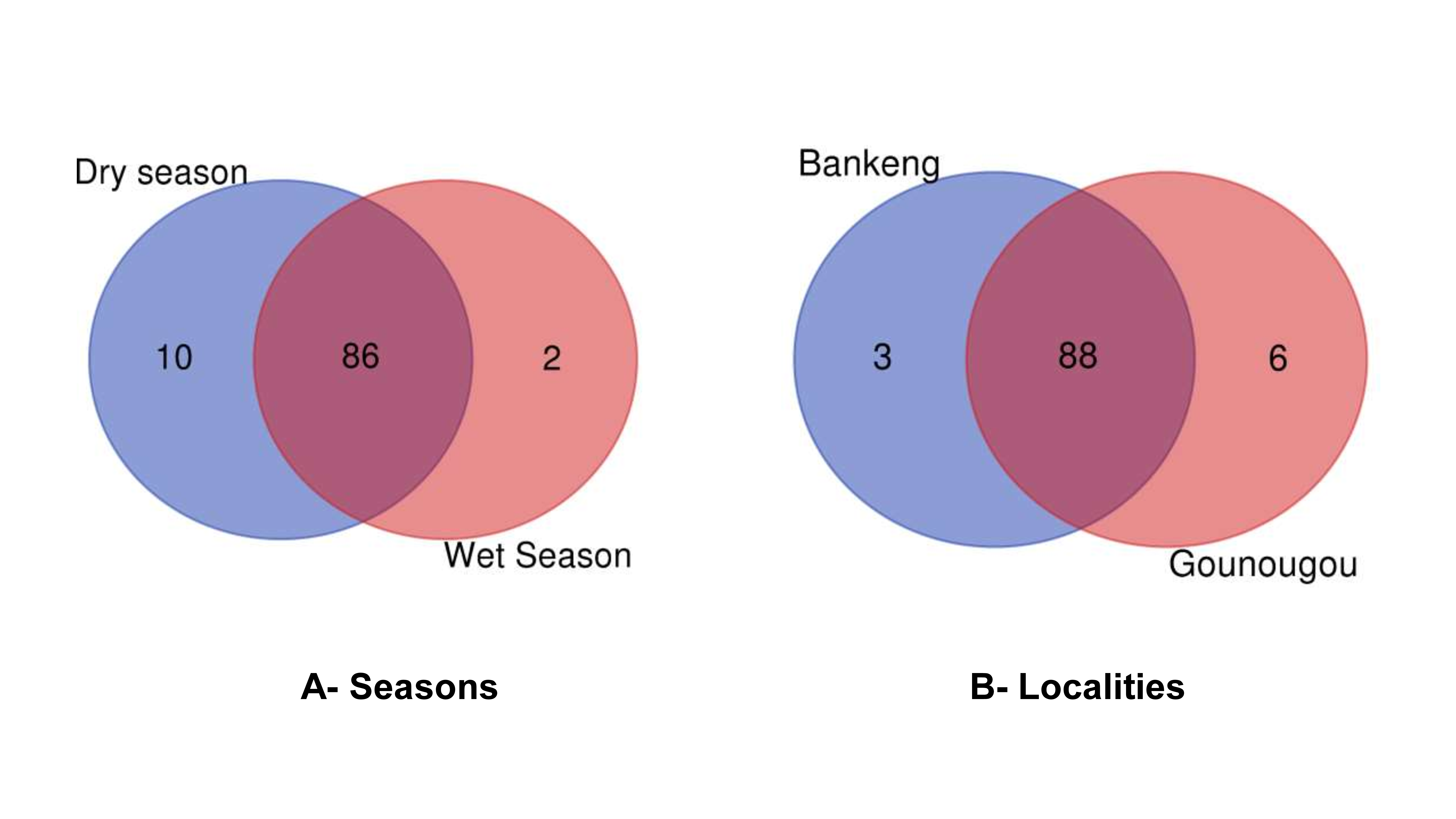

Supplement: Supplementary file 6 — Figure S6. Venn diagrams showing the number of shared or unique bacterial ASVs among dry and wet seasons and among localities annotated to the genus level. (A) Number of unique and shared microbial taxa between seasonal (dry vs. wet). (B) Number of unique and shared microbial taxa between collection areas or Anopheles species (Bankeng vs. Gounougou). [file MVE-36-269-s008.tiff]
